# Comparative genomic analysis of Colistin resistant *Escherichia coli* isolated from pigs, a human and wastewater on colistin withdrawn pig farm

**DOI:** 10.1038/s41598-023-32406-w

**Published:** 2023-03-29

**Authors:** Nwai Oo Khine, Thidathip Wongsurawat, Piroon Jenjaroenpun, David J. Hampson, Nuvee Prapasarakul

**Affiliations:** 1 Center of Excellence in Diagnosis and Monitoring of Animal Pathogens (DMAP), Department of Veterinary Microbiology, Faculty of Veterinary Science, Chulalongkorn University, Bangkok, Thailand; 2 Division of Bioinformatics and Data Management for Research, Research Group and Research Network Division, Faculty of Medicine Siriraj Hospital, Mahidol University, Bangkok, Thailand; 3 School of Veterinary Medicine, Murdoch University, Perth, WA Australia

**Keywords:** Evolution, Genetics, Microbiology, Environmental sciences

## Abstract

In this study, genomic and plasmid characteristics of *Escherichia coli* were determined with the aim of deducing how *mcr* genes may have spread on a colistin withdrawn pig farm. Whole genome hybrid sequencing was applied to six *mcr-*positive *E. coli* (MCRPE) strains isolated from pigs, a farmworker and wastewater collected between 2017 and 2019. Among these, *mcr*-1.1 genes were identified on IncI2 plasmids from a pig and wastewater, and on IncX4 from the human isolate, whereas *mcr-*3 genes were found on plasmids IncFII and IncHI2 in two porcine strains. The MCRPE isolates exhibited genotypic and phenotypic multidrug resistance (MDR) traits as well as heavy metal and antiseptic resistance genes. The *mcr*-1.1-IncI2 and IncX4 plasmids carried only colistin resistance genes. Whereas, the *mcr-*3.5-IncHI2 plasmid presented MDR region, with several mobile genetic elements. Despite the MCRPE strains belonged to different *E. coli* lineages, *mcr-*carrying plasmids with high similarities were found in isolates from pigs and wastewater recovered in different years. This study highlighted that several factors, including the resistomic profile of the host bacteria, co-selection via adjunct antibiotic resistance genes, antiseptics, and/or disinfectants, and plasmid-host fitness adaptation may encourage the maintenance of plasmids carrying *mcr* genes in *E. coli.*

## Introduction

Colistin (polymyxin E) is a high priority antimicrobial that is a treatment of choice for infections with multidrug resistant *Enterobacteriaceae*. The extensive use of colistin in livestock has encouraged the rapid spread of plasmid mediated *mcr* genes encoding colistin resistance. Several reports have suggested that farm animals can be a source of *mcr*-1 that spreads to humans^1,2^. The *mcr-*1 gene is the most common member of the *mcr* gene family and has been found in bacteria from many ecological niches^3^. Multiple plasmid types, particularly the IncX4, IncI2, and IncHI2 plasmids, have been found to contain *mcr*-1 and *mcr-*3 genes^4,5^. Additionally, the use of biocides or other antibiotics may result in co-selection of *mcr* genes and/or cross-resistance to colistin^6,7^. The frequent recovery of *mcr*-positive bacteria, particularly *E. coli*, from humans, animals, and the environment is very problematic. Despite numerous cases of colistin resistant *Enterobacteriaceae* being reported from livestock and humans in Thailand, detailed genomic characterization of *mcr*-positive *E. coli*, particularly amongst livestock isolates, is still limited. In this study, six *mcr-*positive *E. coli* strains with multi-drug resistance traits^8^ were subjected to whole genome sequencing (WGS) and characterization of the plasmids for better understanding of *mcr* genes dissemination between pigs and the farm environment.

## Results

### Genomic characterization of *mcr* positive *E. coli*

The six MCRPE strains submitted for whole genome sequencing had colistin MIC values of 4–8 mg/L and had different PFGE profiles and ST types^8^. These MCRPE strains transferred *mcr*-1 and *mcr*-3 genes to recipient *E. coli* J53 at conjugative transfer frequency rate of 1.7–2 × 10^−4^. Their genome sizes ranged from 4 to 4.8 Mb. Detailed information about the colistin resistant *E. coli*, including their serotypes detected by Serotype finder 2.0 is presented in Table 1. The *mcr*-1.1 gene was detected in three of the six strains, one each from a human (CP52E, 2017), a wastewater sample (CPWW7, 2017) and a pig (CPA1200, 2019), and these showed 100% identity to *mcr*-1 in KP347127, the first *mcr-*1 gene that was identified in China^2^. Unfortunately, the *mcr*-1 gene and other *mcr* genes were not found after in silico analysis in the *E. coli* of wastewater origin from 2018 (CPWWCT), even though it had tested positive previously by PCR and was phenotypically resistant during screening. Seemingly, this strain had lost the *mcr*-1 plasmid during sub-culturing. Analysis of the plasmids in this *E. coli* strain showed that the IncX1 plasmid harboured various mobile genetic elements together with various AMR genes (supplementary Fig. 1).

**Table 1:** Genomic features including sequence type (ST) in MLST, serotype, resistance genes, and virulence gene profiles of the six *E. coli* strains subjected to whole genome sequencing.

Strain	Year	Source	ST	Contig	Location	Serotype	Resistance genes	Virulence genes
CP 52E	2017	Human	515	CP52E-Chromosome	Chromos-ome	O128: H12	*dfrA12, mdf(A)*, *tet(A)*, *sul3, bla*_TEM-1B_ *bla*_CTX-M-14_*, cmlA1* *aph(3'')-Ib, aadA2* *aph(6)-Id, aph(3')-Ia, aac(3)-IId, aadA1,sul1*	*fyuA, gad, irp2,* *terC, hlyE,* T3SS
				pCP52E-IncFIB	Plasmid		*bla*_TEM-1B_	
				pCP52E-IncX4	Plasmid		***mcr-*1.1**	
				pCP52E-ColpVC	Plasmid		–	
CP E35	2017	Pig	10	CPE35-chromosome	Chromos-ome	O101:H9	*tet(A)*	*gad, iss, terC, hlyE*
				pCPE35-IncFIB	Plasmid		*bla*_CMY-2_	
				pCPE35-IncX1	Plasmid		*aadA1, aadA2* *aph(3')-Ia, aph(6)-Id, bla*_TEM-1B_*, cmlA1* *dfrA12, strA, sul3*	
				pCPE35-IncFII	Plasmid		*erm(B),* ***mcr-*3.20**	
				pCPE35-ColE10	Plasmid		–	
				pCPE35-ColPVc	Plasmid		–	
CPWW7	2017	Wastewater	453	CPWW7- chromosome	Chromos-ome	O23:H16	*mdf(A), sitABCD*	*gad, iss, fyuA* *lpfA, astA, hlyE, hlyF,* *terC,* T3SS
				pCPWW7-IncFII	Plasmid		*erm(B), qnrS(1)*	
				pCPWW7-IncI2	Plasmid		***mcr-*1.1**	
				pCPWW7-IncY	Plasmid		*tet(A), aadA2, sul1, sul3, qacE, dfrA12*	
				pCPWW7-unnamed plasmid	Plasmid		*bla*_TEM-1B_*, aph(6)-Id, aph(3')-Ia, aph(3'')-Ib* *aadA22, aac(3)-IId, sul2, catA2*	
CPF6	2018	Pig	3944	CPF6-chromosome	Chromos-ome	O8:H2	*mdf(A)*	*fyuA, gad, terC* *traT, hlyE* T3SS
				pCPF6-IncHI2	Plasmid		*aac(3)-IId, aadA1* *aadA2, aph(3'')-Ib* *aph(3')-Ia, aph(6)-Id* *bla*_CTX-M-55_*, cmlA1* *dfrA12, ****mcr-*3.2***, qacC, qnrS1, sul3, tet(A)*	
				pCPF6-IncFII	Plasmid		***mcr-*3.5**	
				pCPF6-IncFIB	Plasmid		–	
				pCPF6- IncI1	Plasmid		–	
				pCPF6-IncX1	Plasmid		–	
CPWWCT	2018	Waste water	453	CPWWCT-chromosome	Chromosome	O70:H10	*sul2, sul1, mdf(A),* *bla*_CTX-M-63_*, dfrA12, aadA2, qnrS1, qacE*	*gad, iss, terC, hlyE,* T3SS
				pCPWWCT-unnamed plasmid	Plasmid		*sul1, aph(3')-Ia, aadA2, qacE*	
				pCPWWCTp0111	Plasmid		*bla*_TEM-1C_	
				pCPWWCT-IncI1	Plasmid		–	
				pCPWWCT-IncQ1	Plasmid		–	
				pCPWWCT-ColpVC	Plasmid		–	
				pCPWWCT-IncX1	Plasmid		*tet(M), tet(A), erm(B), qnrS1*	
CP A1200	2019	Pig	New ST	CPA1200-chromosome	Chromosome	08:H2	*mdf(A)*	*iss, ompT, terC, hlyE, hlyF*
				pCPA1200-IncFIB-FIC	Plasmid		*aph(6)-Id, aph(3'')-Ib,* *bla*_TEM-1B_*, tet(A), sul2, sitABCD*	*iss, iucC, iutA, ompT, sitA, traT* *tsh*
				pCPA1200-IncI2	Plasmid		***mcr-*1.1**	
				pCPA1200-IncI1	Plasmid		–	
				pCPA1200-IncQ1	Plasmid		–	
				pCPA1200-IncFII(pCoo)	Plasmid		–	

Significant values are in bold.

**Figure 1:**
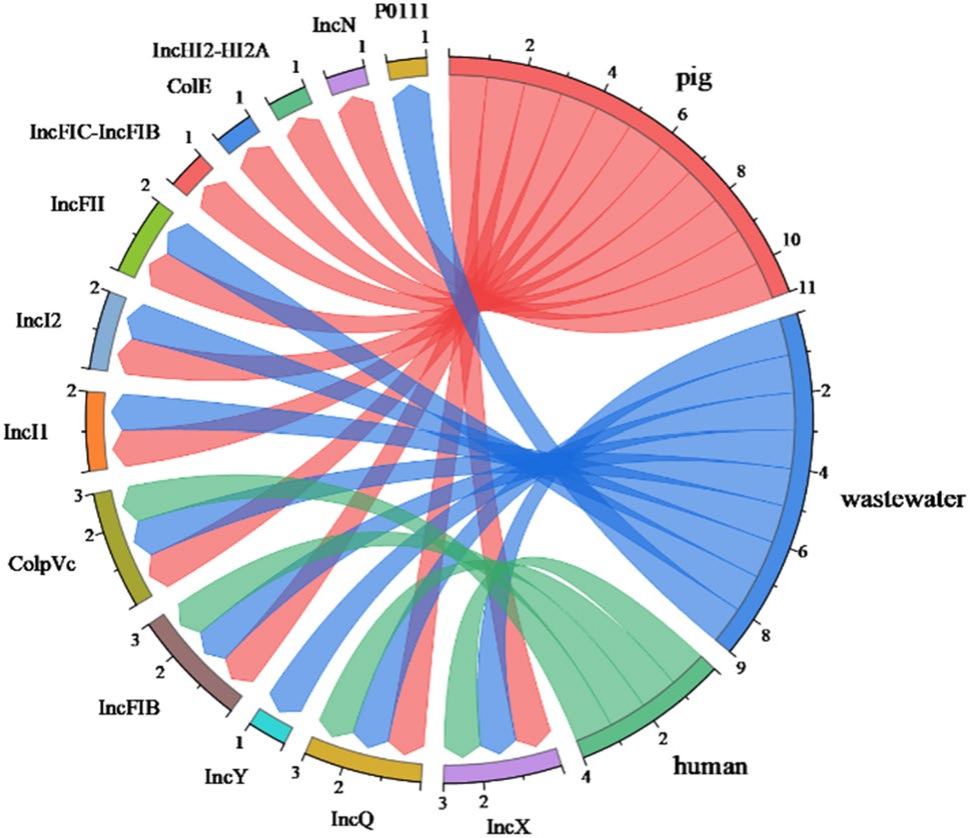
Chord diagram showing the various plasmid replicon types detected from the six colistin resistant *E. coli* strains.

Two of the porcine strains were *mcr*-3 positive: CPE35 (Pig, 2017) contained *mcr*-3.2 on the IncFII plasmid while CPF6 (Pig, 2018) contained *mcr*-3 variants *mcr*-3.2 and *mcr*-3.5 on IncHI2 and IncFII, respectively. None of the MCRPE strains carried *mcr* genes on their chromosomes. All the *mcr-*1.1 genes found in this study were located either on IncI2 or IncX4 plasmids. Several plasmids, including both phenotypically known and unnamed plasmids were detected in all MCRPE strains. Moreover, all the plasmid replicon types detected in the MCRPE strain of human origin were also found in strains from pigs and wastewater (Fig. 1).

### Antibiotic resistance genes and virulence genes

A total of 31 ARGs were identified amongst the six strains, with those encoding resistance to aminoglycosides, beta-lactams, cephalosporins, fluoroquinolones, trimethoprim, macrolides, chloramphenicol, sulfonamides and tetracyclines (Table 1). Genes encoding resistance to the disinfectant; hydrogen peroxide and quaternary ammonium compounds were detected in the two porcine *E. coli* strains collected from 2018 and 2019 and in both strains of wastewater origin. Apart from the plasmids pCPE35-IncFII (pig, 2017) and pCPF6-IncHI2 (pig, 2018), the other plasmids harbouring *mcr* in this study contained only colistin resistance genes. Moreover, the genomes of four MCRPE strains (except for CPE35 and CPWW7) contained genes conferring resistance to the heavy metals copper, silver and zinc (supplementary Table 1). On the other hand, CPWW7 carried mercury resistance genes on the plasmid as well as various aminoglycosides resistance genes. Genes encoding a multidrug resistance efflux pump including *emrD, mdtM* and *mdfA* were also detected on the chromosome of MCRPE strains.

All six MCRPE carried genes encoding various virulence factors (Table 1), with the majority of these being present on the chromosomes. Virulence genes of different pathotypes encoding virulence factors such as adherence factors, flagellar associated proteins, fimbrial adhesin proteins, *hlyE, hlyF* (haemolysin), type III secretion system related factors, toxins (*astA*, enteroaggregative heat-stable toxin, EAST-1), and siderophore receptors (*fyuA*) were detected. The most common virulence genes detected were associated with type III secretion systems, adhesion, and haemolysis (*hlyE*).

### Genomic insights into the *mcr-*1 *and mcr-*3 positive *E. coli* strains

**Figure 2:**
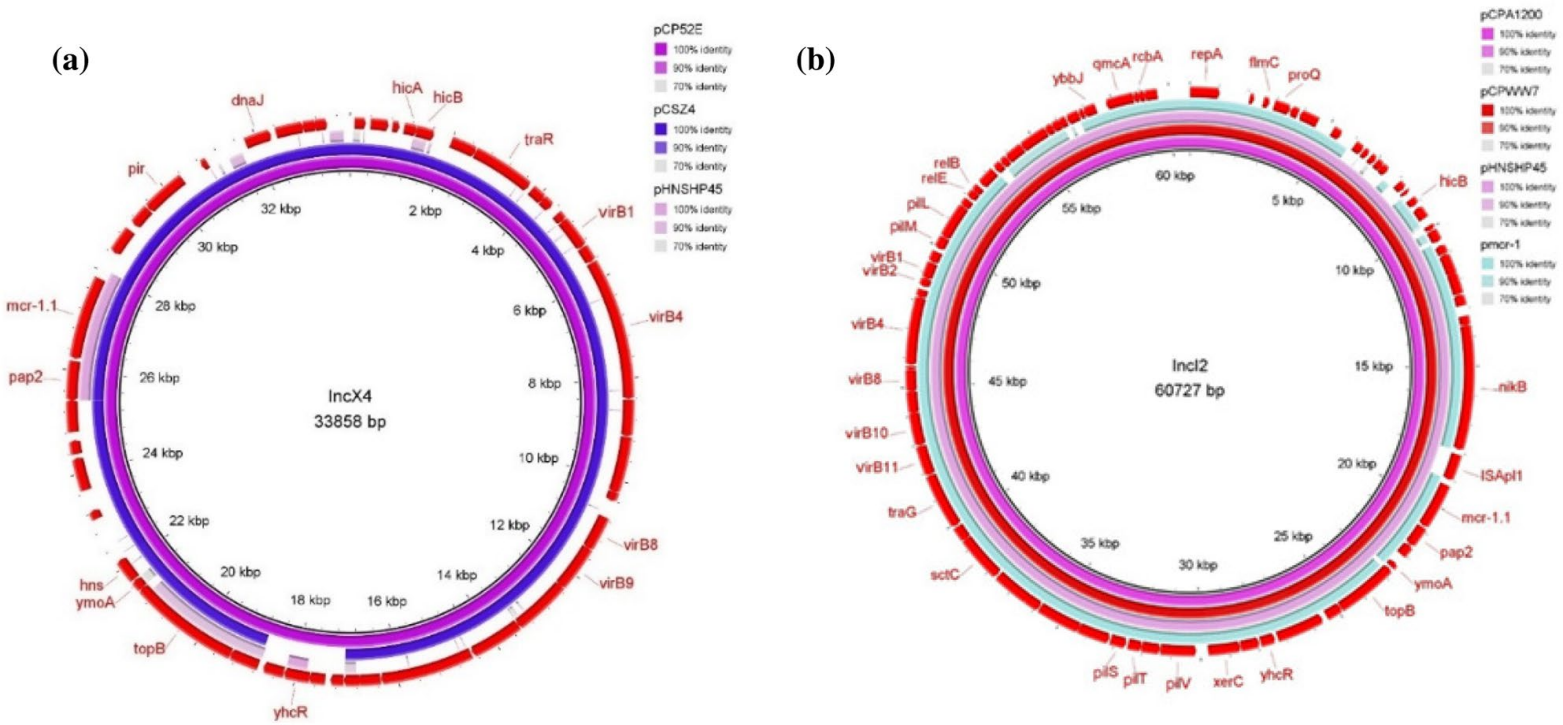
Sequence alignment and circular comparison of *mcr*-1 plasmids. Panel (**a**): Sequence alignment of pCP52E-IncX4 (human, 2017) plasmid with pCSZ4 (GenBank no. KX711706) and pHNSHP45 (GenBank no. KP347127). The outer circle with red arrows denotes annotation of the plasmid pCP52E-IncX4. Panel (**b**): Sequence alignment of pCPA1200-IncI2 (pig, 2019) plasmid with pCPWW7-IncI2 (wastewater, 2017) and pHNSHP45 (GenBank no. KP347127) and pmcr1_IncI2 (GenBank no. KU761326). The outer circle with red arrows denotes annotation of the plasmid pCPA1200-IncI2.

The *mcr*-genes were detected in IncX4 (n = 1), IncHI2 (n = 1), IncI2 (n = 2) and IncFII (n = 2). The ~ 33 kb pCP52E-IncX4 plasmid harboured by *E. coli* of human origin did not contain resistance genes other than *mcr*-1.1 on the same plasmid, and no ISApl1 elements were found flanking the *mcr-*1.1 gene. Typical plasmid backbone features such as genes encoding type IV secretion system (T4SS) proteins and toxin-antitoxin system components (HicA-HicB) were present. A structural comparison of pCP52E-IncX4 against other reference plasmids that contained *mcr*-1 is shown in Fig. 2a. The IncX4-*mcr*-1 plasmid in this study shared 95% coverage and 99.95% identity with pCSZ4 (GenBank no. KX711706) from an *E. coli* isolate of porcine origin from China.

**Figure 3:**
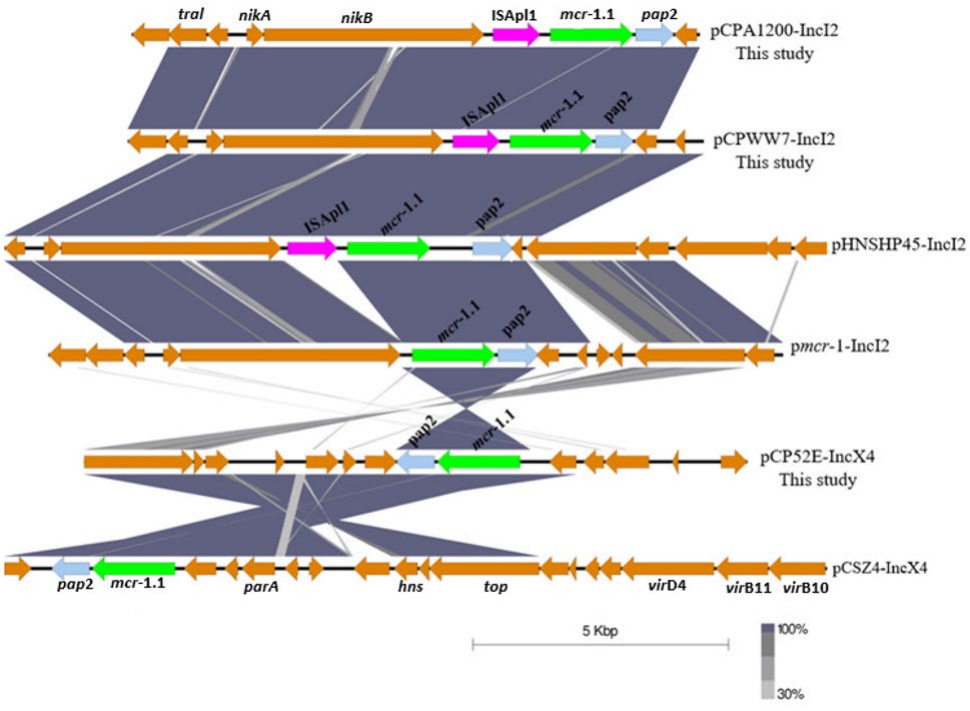
Comparison of the genetic environment of the *mcr*-1.1 gene from MCRPE in this study with references plasmids. The grey area indicates the blast identities, and the percentage of identity is indicated in the legend. Open arrows represent coding sequences (green for *mcr*-1.1, blue for PAP2, purple for ISApl1 and yellow for other genes). The arrow size is proportional to the gene length. The image was generated using EasyFig with default parameters.

The *mcr*-1.1-IncI2 plasmids of pCPA1200-IncI2 (pig, 2019) and pCPWW7-IncI2 (wastewater, 2017) shared high similarity, including 99% coverage and 100% identity. A structural comparison of pCPA1200-IncI2 with pCPWW7-IncI2 and pHNSHP45 (IncI2 plasmid reported from China) is shown in Fig. 2b. Moreover, these IncI2 plasmids contained numerous conjugation related genes such as T4SS, and pilus modification and conjugative transfer system protein genes. The ~ 60 kb IncI2-*mcr*-1.1 plasmids showed the same genetic structure as ISApl1-*mcr*-1-pap2, with loss of downstream ISApl1. A comparison of the genetic environment of the *mcr*-1.1 cassette from IncI2 and IncX4 plasmids from this study and the references plasmids is presented in Fig. 3.

**Figure 4:**
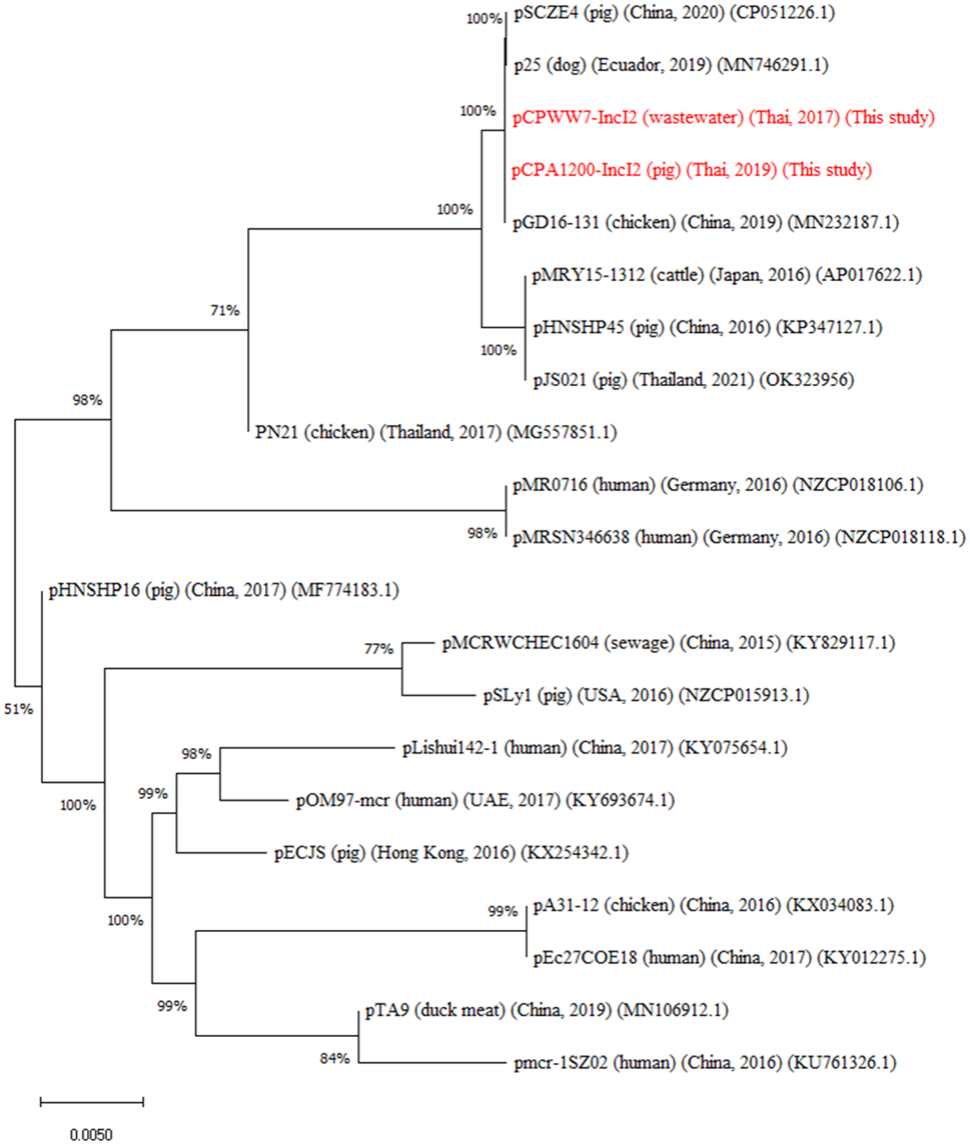
Phylogenetic analysis of 22 *mcr*-1-harbouring IncI2 plasmids. The *mcr-*1-carrying IncI2 plasmids from this study (red colour) pCPWW7-IncI2 from wastewater and pCPA1200-IncI2 from a pig and other *mcr-*1-carrying IncI2 plasmids of *E*. *coli* deposited in the GenBank database. Sequences were aligned using MAFFT with default values and the phylogenetic tree was constructed by using the neighbour-joining method with the MEGA 10 software.

The phylogenetic tree constructed for the *mcr-*1.1-harbouring IncI2 plasmids of different origins identified six distinct clades (Fig. 4). The IncI2 plasmids from this study, pCPWW7-IncI2 and pCPA1200-IncI2, were in the same clonal lineage and related to the plasmid pSCZE4 from a pig (China), p25 from a dog (Eucador) and pGD16-131 from a chicken (China). Moreover, IncI2 plasmids in this study also were found to be clonally related to pHNSHP45 (pig, China), pMRY15-1312 (cow, Japan) and pJS021 (pig, Thailand), showing that highly related *mcr-*1-IncI2 plasmids from different sources are distributed globally.

**Figure 5:**
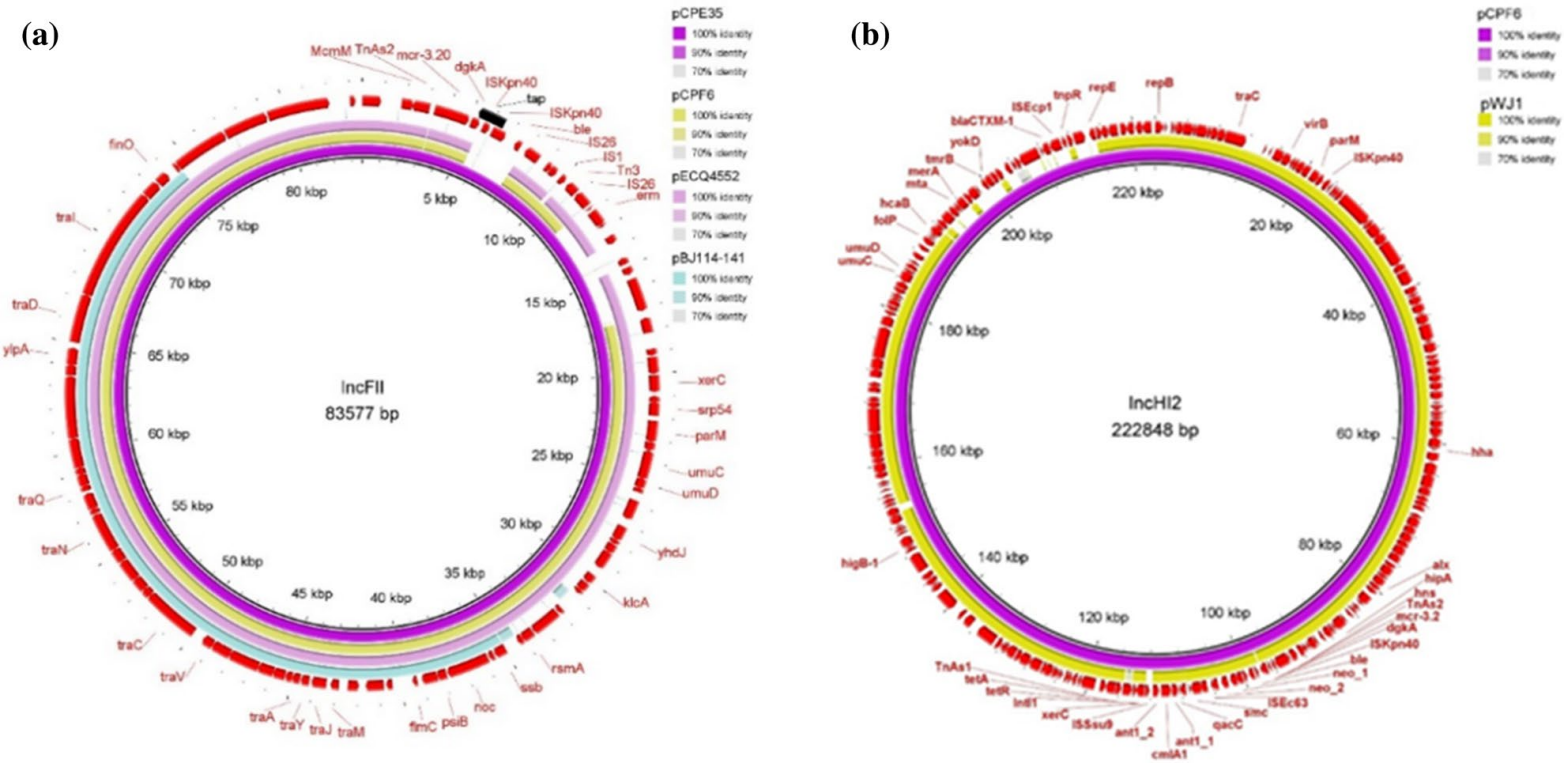
Sequence alignment and circular comparison of *mcr*-3 plasmids. Panel (**a**): Sequence alignment of pCPE35-IncFII (pig, 2017) *mcr*-3.2 plasmid with pCPF6-IncFII (pig, 2018) *mcr*-3.5 plasmid (this study) and the reference plasmids pECQ4552 (GenBank no. CP077064.1) and pBJ114-141 (GenBank no. MF679146). The outer circle with red arrows denotes annotation of the plasmid pCPE35-IncFII. The black bar represents the multidrug efflux pump (Tap) from pCPF6. Panel (**b**): Sequence alignment of pCPF6-IncHI2 (pig, 2018) *mcr*-3.2 plasmid with pWJ1 (GenBank no. KY924928). The outer circle with red arrows denotes annotation of the plasmid pCPF6-IncHI2.

**Figure 6:**
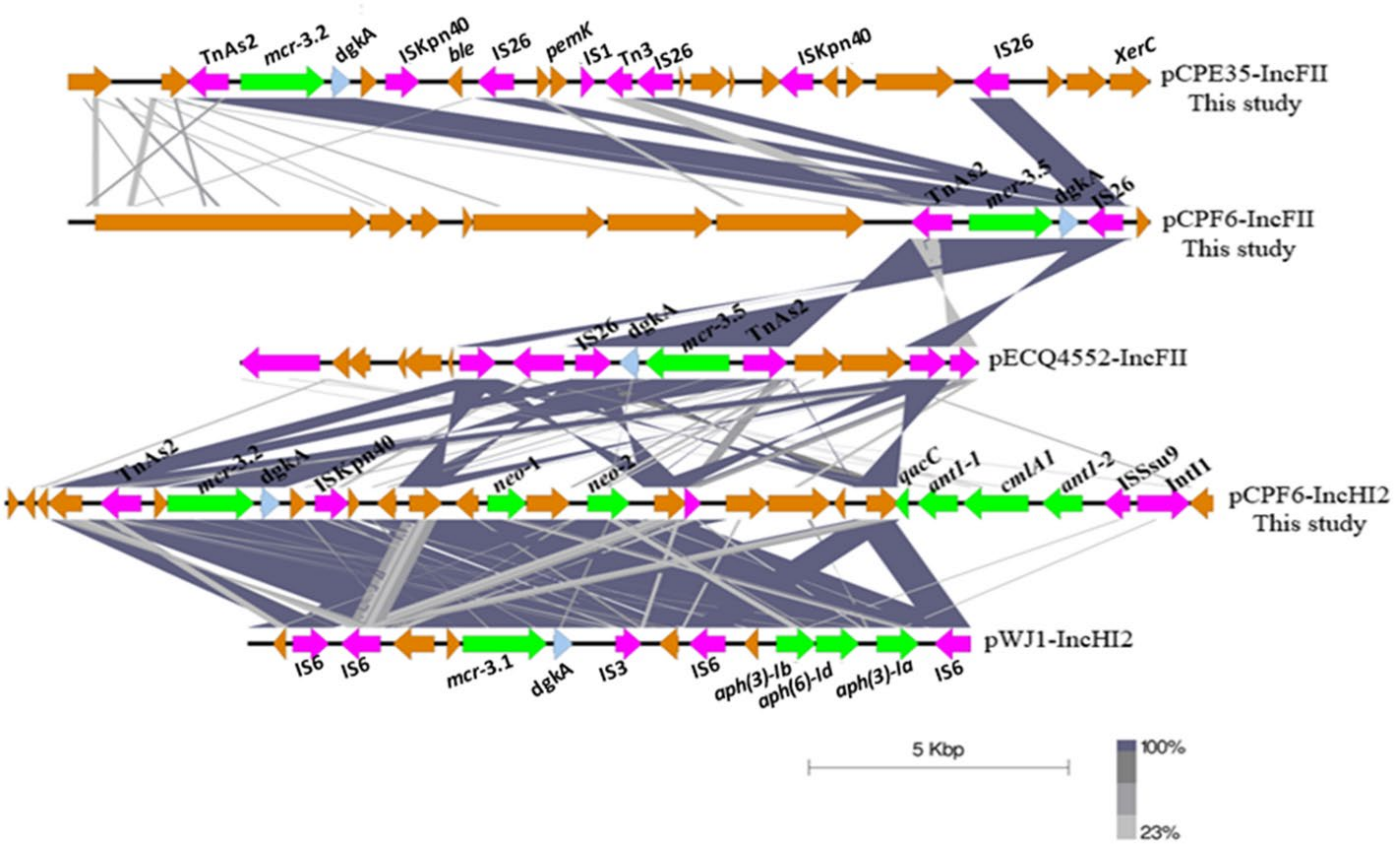
Comparison of the genetic environment of *mcr*-3 genes from MCRPE isolates from this study with references plasmids. The grey area indicates the blast identities, and the percentage of identity is indicated in the legend. Open arrows represent coding sequences (green for antimicrobial resistance genes, blue for dgkA (orf), purple for mobile genetic elements and yellow for other genes). The arrow size is proportional to the gene length. The image was generated using EasyFig with default parameters.

**Figure 7:**
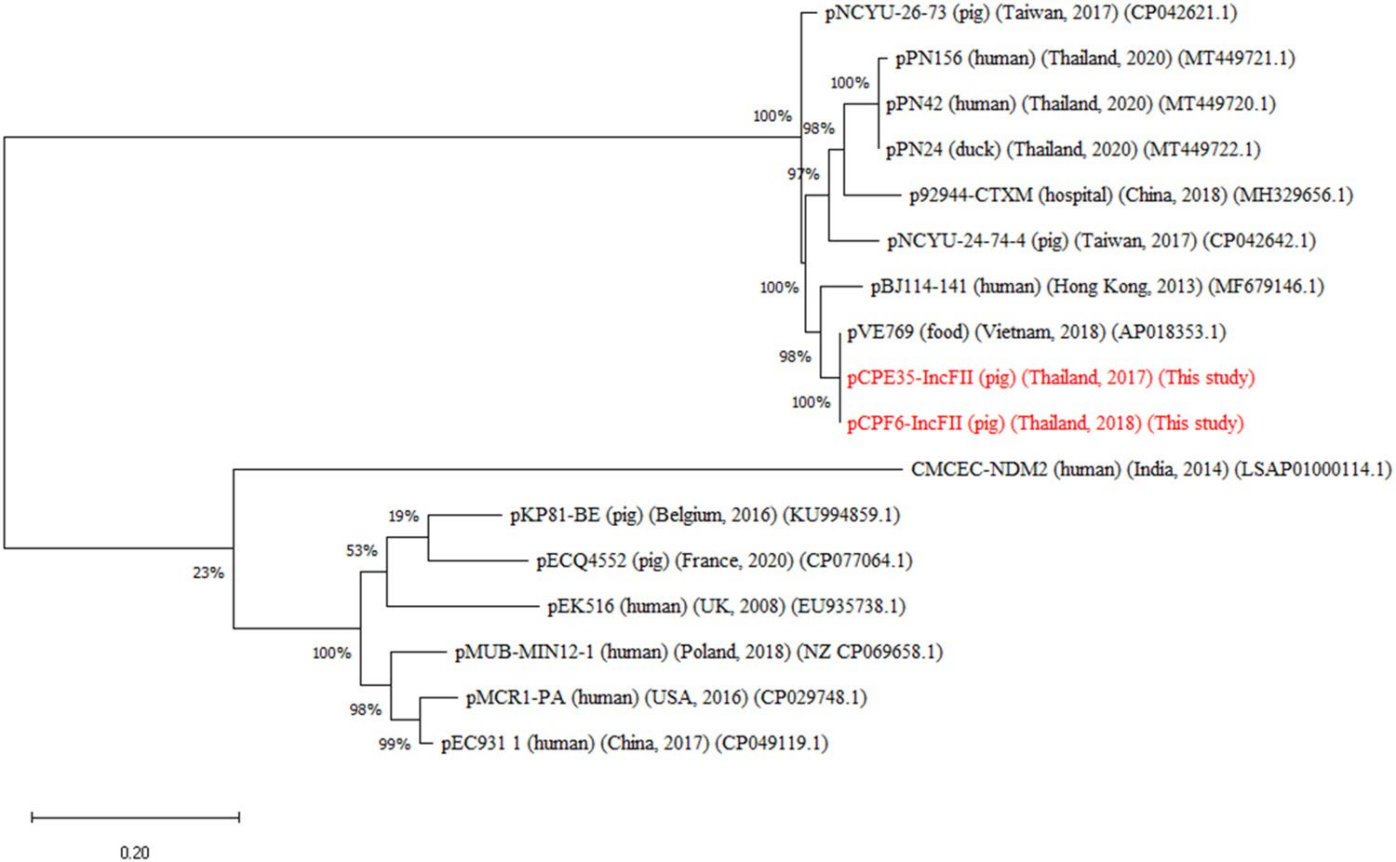
Phylogenetic analysis of *mcr*-3 IncFII plasmids from this study with 15 IncFII plasmids of *E. coli* deposited in the GenBank database. The *mcr*-3-carrying IncFII plasmids from this study pCPE35-IncFII and pCPF6-IncFII from pigs are shown in red. Sequences were aligned using MAFFT with default values and the phylogenetic tree was constructed by using the neighbour-joining method with the MEGA 10 software.

The ~ 83 kb IncFII plasmids which carried *mcr*-3.2 in pCPE35-IncFII (pig, 2017) and *mcr-*3.5 in pCPF6-IncFII (pig, 2018) had 92% coverage with 99% identity. These IncFII plasmids harboured conjugation related transfer protein genes (*tra*). Moreover, the pCPE35-IncFII plasmid contained genes encoding microcin producing protein (McmM) while pCPF6-IncFII contained genes for a MDR efflux pump protein (Tap) (Fig. 5a). The pCPF6-IncHI2 (pig, 2018) plasmid showed high identity with the IncHI2 plasmid pWJ1 (GenBank no. KY924928) from a Chinese porcine *E. coli* isolate (Fig. 5b). Genetic arrangements in the vicinity of *mcr*-3.2 comprised TnAs2–*mcr*-3.2–dgkA–ISKpn40. In contrast, *mcr*-3.5 on the pCPF6-IncFII plasmid was flanked by TnAs2-*mcr*-3.5-dgkA-IS26 (Fig. 6). The plasmid pCPF6-IncHI2 contained multiple resistance genes against aminoglycosides, tetracycline and extended spectrum beta lactamases (ESBL). Moreover, a disinfectant resistance gene (*qacC*) and an integron (*Intl*1) were also detected downstream of the *mcr*-3.2 gene on the same plasmid. A comparison of the genetic environment surrounding the *mcr-*3 cassette in the IncFII and IncHI2 plasmids from this study and the reference plasmids are shown in Fig. 6. A phylogenetic tree constructed based on core genome sequences of *mcr*-3 carrying IncFII plasmids from this study and from 15 IncFII plasmids deposited in the GenBank database identified two distinct subclades, as shown in Fig. 7. Interestingly, the IncFII plasmids from this study together with IncFII plasmids from Asian countries (China, Hong Kong, Vietnam, Thailand) were found to be closely related and branched as a group in the phylogenetic tree. On the other hand, IncFII plasmids from Europe and the USA were different and were found in distinct subclades from the pCPF6-IncFII and pCPE35-IncFII plasmids of this study. The accession numbers and detailed information for the references plasmids is provided in (supplementary Table 2).

## Discussion

The six *E. coli* strains selected for WGS in this study were found to have resistance genes against various antibiotic classes in both their chromosomes and plasmids. Genes conferring resistance to disinfectants and biocides were also detected in the MCRPE strains. Biocides are often present in agricultural products and feed additives, and their stability in the environment acts to prolong exposure and selective pressure on bacteria^9^. Notably, bacterial resistance to the above compounds could favour co-selection and co-expression of various antibiotic resistance genes that also may be present^10^.

The MCRPE in this study also possessed various virulence genes. Notably, strain CPE35 from a pig belonged to ST10 with serotype O101: H9, which has been reported to be associated with animal and human disease^11^. This serotype has been reported in Shiga toxin-producing *E. coli* (STEC) from humans and in enterotoxigenic *E. coli* (ETEC) from calves with diarrhoea in Europe^12,13^. Serotype O128: H12, which was identified in the MCRPE from a human (CP52E) in this study, has been associated with ETEC as well as in enteropathogenic *E. coli* (EPEC)^12^. These findings support the likelihood that healthy pigs may be carriers for MDR bacteria which also may harbour various virulence genes, and which may be disseminated to humans.

The epidemic plasmids IncI2 and IncX4 were the source of the *mcr*-1.1 genes identified in this study. IncI2 plasmids largely have been detected in various *mcr*-1 cases from different hosts around the world^14^. On the other hand, the IncX4 plasmid type has been reported as the dominant *mcr*-1-carrier in isolates from healthy humans in China^3^ and in carbapenem and *mcr*-1 co-carrying *Enterobacteriaceae* from clinical patients across Thailand^15^. The IncX4 plasmids have been reported to be genetically less variable and relatively smaller than IncI2 plasmids^16,17^. IncI2 replicon type plasmids are known to have the strongest competitive and fitness advantage in the host bacterium when compared to other plasmid types such as IncHI2 or IncX4 plasmids^14,18^. These considerations also applied in the current study where the *mcr*-1-IncI2 plasmid was still found in *E. coli* isolates recovered long after the colistin ban was implemented at the start of 2017. A previous study also found that *mcr*-1 bearing IncI2 plasmids were present in *E. coli* from fattening pigs that had no exposure to colistin^14^. It was noteworthy that the IncI2 plasmids contained more conjugation transfer system genes than genes associated with replication, which suggests that they are more adapted to spread from host to host than to undergo replication^19^. Therefore, the absence of antibiotic selective pressure might have a neglectable effect on the persistence or conjugative rate of such plasmids that have low fitness cost.

In comparison, *mcr*-3 genes were detected on IncFII and IncHI2 plasmids. The *mcr*-3 gene was first identified in a IncHI2 plasmid in China^5^, and *mcr*-3 mediated IncP and IncFII plasmids previously have been reported in *E. coli* in Thailand^20^. The MDR plasmid pCPF6-IncHI2-*mcr*-3.2 (pig, 2018) from this study contained resistance genes against tetracycline, aminoglycosides, chloramphenicol, cephalosporin, disinfectant, as well as colistin. Various antimicrobial resistance genes located on the same plasmid could enhance the persistence and co-selection of *mcr* genes^21^. Therefore, aside from colistin withdraw, continual monitoring of other antimicrobial use during the pig production cycle is needed to improve control of colistin resistance in pig farms.

The occurrence of highly similar plasmids in different lineages of MCRPE isolates collected from different sources and at different times implies that the associated plasmids have been widely disseminated through the farm. It is concerning that colistin resistance plasmids could persist with relative ease among genotypically distinct *E. coli* strains from different niches, and it implies that the transmission of these plasmids might be extremely hard to control. Moreover, *mcr*-1 is mobilized by a composite transposon called Tn6330, where the *mcr*-1 with a putative open reading frame (PAP2 like protein) is flanked by two ISApl1 insertion sequences^22^. In the IS30 family, ISApl1 performs a ‘copy-out, paste-in’ mechanism and is highly active^23^. Therefore, the *mcr*-1 genes from *E. coli* recovered from pigs and wastewater in this study were mobilizable and able to be persisted even after 2 years without colistin exposure. In agreement with our results, significantly more *mcr*-1 cases with attached insertion sequence ISApl1 have been found amongst animal isolates than in human isolates^24^. These findings are consistent with animals being a primary source for *mcr*-1 bearing bacteria that are transmitted to humans.

In this study, there were too few isolates examined to determine which of the *mcr* genes were most likely to persist after colistin withdrawal. A previous in vitro experiment showed that plasmids carrying *mcr*-3 have greater stability than *mcr*-1 plasmids in the absence of colistin, with *mcr*-3 having a lower fitness cost^25^. On the other hand, another study found that certain *E. coli* strains were more likely to eliminate *mcr*-3 genes than *mcr-*1 genes in vitro, with or without exposure to colistin^16^. Therefore, the persistence and fitness cost of the *mcr*-1 and *mcr*-3 genes in bacteria might differ depending upon the plasmid as well as on the host genetic background. In previous studies, *mcr*-3.2 positive *E. coli* of bovine origin^26^ and *mcr*-3.1 bearing isolates of porcine origin^5^ were found to have genetic environment TnAs2-*mcr*-3.2-dgkA-ISKpn40, which occurred on both IncFII and IncHI2 plasmids in this study. ISKpn40 belongs to the IS3 family and was first identified in an *E. coli* strain from a pig, whereas the IS6 family of IS26 which was detected on the *mcr*-3.5-pCPF6-IncFII plasmid facilitates mobilization of resistance genes in Gram-negative bacteria^27^. Although the genetic environments of *mcr*-3 determinants are variable, the core structure of TnAs2-*mcr*-3-dgkA, accompanied with other mobile elements or resistance genes, is highly conserved^16^.

A previous in vitro study found that in the absence of antibiotic selective pressure, IncFII plasmids were unstable and outcompeted by plasmid-free cells^14^. Notably, the cost of the plasmid is increased according to its metabolic load, such as expression of biomolecules or energy-rich compounds, as well as introduction of an efflux pump^28^. This is consistent with our results where IncFII plasmids encoded either bacteriocin producing proteins or efflux pump proteins. On the other hand, the IncHI2 plasmid found in this study was a MDR plasmid accompanied by several mobile genetic elements. Such kinds of MDR plasmid may present a fitness burden and may be prone to deletion of the *mcr*-3 gene or the whole plasmid from the bacteria.

The metabolic cost of a plasmid increases relatively with increasing levels of resistance genes on the plasmid and the level of their phenotypic expression rather than on the size of the plasmid itself^29–31^. This phenomenon could be related to our finding that the *mcr*-1 bearing plasmids were colistin mono-resistant plasmids with high stability traits. Moreover, the moderate MICs (4–8 mg/L) for colistin, and the fitness benefit expressed by *mcr*-1-bearing IncI2 and IncX4 plasmids further helps to explain their long-term persistence in the farm after colistin withdrawal. Hence, not only cessation of colistin selective pressure, but also the characteristics of *mcr* bearing plasmids and co-selection by other antibiotic usage and farm management need to be considered when attempting to control colistin resistant bacteria in the population.

In conclusion, the *mcr* bearing plasmids could still be transmitted between hosts on the farm for some time after colistin selective pressure was removed. According to our findings, even without antibiotic selective pressure, resistance plasmids with little or no fitness burden on the bacterial hosts could persist in the population. Moreover, the presence of resistance genes against various antimicrobials and disinfectants could help to co-select for colistin resistant bacteria. Undoubtedly, the need to minimize the use of antibiotics as well as continuous monitoring of the antimicrobial resistance profiles of bacteria in livestock farms are essential for AMR control.

## Materials and methods

### Sampling and strain selection

The strains studied were obtained in a previously reported investigation conducted in the central region of Thailand^8^. In that study MCRPE isolates (n = 170) from pigs, wastewater, and workers on a pig farm where routine prophylactic colistin use had been withdrawn at the start of 2017 were obtained over a three-year period and longitudinally monitored. Six of these multi-drug resistant MCRPE strains with different clonal types based on pulsed field gel electrophoresis and similar plasmid replicon types found by PCR were selected for the current molecular study. The strains comprised three from pigs, two from wastewater and one from a farm worker. The strains were isolated in mid-2017 (n = 3), 2018 (n = 2) and 2019 (n = 1). A more detailed description of the origin of the six strains is presented in Table 1.

### Bacterial identification and antimicrobial susceptibility testing

The MCRPE strains were identified as *E. coli* using IMViC biochemical tests and MALDI-TOF MS^32^. The *mcr-*1-5 genes were detected by multiplex PCR, as previously reported^33^. The minimum inhibitory concentration (MIC) for colistin was determined by using the broth microdilution technique, and an MIC value of ≥ 4 mg/L was considered to indicate colistin resistance (CLSI, 2021). Antimicrobial susceptibility testing for *mcr* positive *E. coli* strains was performed by using the AST-GN 38 test kit in a Vitek2 apparatus (BioMérieux, France)^34^.

### Plasmid conjugation

Conjugation was performed by broth mating technique to confirm *mcr* genes were located on conjugative plasmids, and their transferability rate^35^. The *E. coli* J53 strain which is resistant to sodium azide (MIC > 512 mg/L) and susceptible to colistin (MIC < 2 mg/L) were applied as recipient strain. Transconjugants were cultured on LB agar (Oxoid) plates containing colistin (2 mg/L) and sodium azide (100 mg/L). The presence of *mcr* genes in transconjugants were determined by PCR as described above.

### DNA preparation and whole genome sequencing

The genomic DNA of the *E. coli* strains was extracted by using the ZymoBIOMICS DNA Miniprep Kit (Zymo Research, USA) according to the manufacturer’s instructions. The extracted DNA was subjected to quantity checking using a Qubit Fluorometer. The samples then were submitted for sequencing using the Illumina NovaSeq PE150 platform and MinION (Oxford Nanopore Technologies for long read sequencing).

### Sequence analysis

The paired-end reads were quality filtered to remove adapters and low-quality sequences with quality scores < 30 by using Trimmomatic v.0.36.5^36^. The related bioinformatic analyses were performed on the European Galaxy server (https://usegalaxy.eu). Reads assembly were perform by using the Unicycler hybrid assembly (Galaxy Version 0.4.8.0) with default settings^37^. Sequences were analyzed for species identification (KmerFinder 2.1), Multilocus Sequence Type (MLST 1.6), virulence factors (VirulenceFinder 1.2), antimicrobial resistance (ResFinder 2.1), plasmids (PlasmidFinder 1.2) and mobile elements finders (v1.0.3) using the Center for Genomic Epidemiology (CGE) pipeline^26^. Acquired antimicrobial resistance genes (ARGs), and *E. coli* virulence factors were also identified using ABRicate on the Galaxy server (Galaxy Version 1.0.1). The databases used in this platform were CARD Resistance Gene Identifier^38^ and ARG-ANNOT (Antibiotic Resistance Gene-ANNOTation)^39^ databases, while for virulence genes the VFDB databases were used^40^. The genomes of MCRPE isolates were annotated by the NCBI Prokaryotic Genomes Annotation Pipeline (PGAP) and Prokka (Prokaryotic genome annotation) (Galaxy Version 1.14.6)^41^. Screening for biocide resistance genes was carried out on the BacAnt server^42^ using the antibacterial biocide and metal resistance gene databases ResDB and BacMet^43^.

Plasmid sequences were obtained using plasmid finder and annotated by Prokka (Galaxy Version 1.14.6)^41^. The contigs of *mcr* variants were compared with reference sequences using BLAST. The annotated plasmids carrying *mcr* genes were compared with reference plasmids belonging to the same Inc groups using BLAST ring image generator (BRIG)^44^, and the genetic context of *mcr*-1 and *mcr*-3 contigs were visualized by using Easyfig (http://mjsull.github.io/Easyfig/)^45^. All the reference plasmids and sequences used in the study were recovered from the NCBI database.

Since highly similar *mcr* bearing plasmids of the IncI2 and IncFII plasmids were detected in isolates from pigs and wastewater recovered in different isolation years, a phylogenetic comparison was performed on the IncI2 and IncFII plasmids from this study with reference plasmid sequences from GenBank. A phylogenetic tree of the *mcr*-1.1-carrying IncI2 plasmids from this study was constructed by comparing with 20 *mcr*-1-carrying IncI2 plasmids deposited in the GenBank database. The sequences were aligned using MAFFT (Galaxy Version 7.505 + galaxy0)^46^ and a phylogenetic tree was obtained by using the neighbour-joining method in MEGA 10 software using1000 times bootstrap values. Likewise, phylogenetic trees of the two *mcr*-3 carrying IncFII plasmids were generated by comparing with 15 *E. coli* IncFII plasmids deposited in the GenBank database.

### Nucleotide sequence accession numbers

The complete nucleotide sequences of the six MCRPE strains CP52E, CPE35, CPWW7, CPF6, CPWWCT and CPA1200 were deposited in GenBank under the accession numbers CP075731, CP075722, CP075716, CP075737, CP076575 and JAHKSR000000000, respectively. The plasmids containing *mcr* genes were: pCP52E-IncX4 (accession number-NZ_CP075733.1), pCPA1200-IncI2 (accession number- JAHKSR010000004.1), pCPWW7-IncI2 (accession number- NZ_CP075719.1), pCPE35-IncFII (accession number- NZ_CP075741.1), pCPF6-IncFII (accession number- CP075741.1) and pCPF6-IncHI2 (accession number- CP075738.1).

### Supplementary Information

Supplementary Information.

## Data Availability

The datasets generated and/or analysed during the current study are available in the National Library of Medicine repository BioProject: PRJNA735516 and PRJNA731849.

## References

[1] Cheng P (2021). Prevalence and characteristic of swine-origin *mcr*-1-positive *Escherichia coli* in Northeastern China. Front Microbiol.

[2] Liu YY (2016). Emergence of plasmid-mediated colistin resistance mechanism *mcr-*1 in animals and human beings in China: A microbiological and molecular biological study. Lancet Infect Dis.

[3] Shen C (2020). Dynamics of *mcr-*1 prevalence and *mcr-*1-positive *Escherichia coli* after the cessation of colistin use as a feed additive for animals in China: A prospective cross-sectional and whole genome sequencing-based molecular epidemiological study. The Lancet Microbe.

[4] Wang Q (2017). Expanding landscapes of the diversified *mcr-*1-bearing plasmid reservoirs. Microbiome.

[5] Yin W (2017). Novel plasmid-mediated colistin resistance gene *mcr-*3 in *Escherichia coli*. MBio.

[6] Wand ME, Bock LJ, Bonney LC, Sutton JM (2017). Mechanisms of increased resistance to chlorhexidine and cross-resistance to colistin following exposure of *Klebsiella pneumoniae* clinical isolates to chlorhexidine. Antimicrob. Agents Chemother..

[7] Xu F, Zeng X, Hinenoya A, Lin J (2018). *mcr*-1 confers cross-resistance to bacitracin, a widely used in-feed antibiotic. mSphere.

[8] Khine NO (2022). Longitudinal monitoring reveals persistence of colistin-resistant *Escherichia coli* on a pig farm following cessation of colistin use. Front. Vet. Sci..

[9] Seiler C, Berendonk TU (2012). Heavy metal driven co-selection of antibiotic resistance in soil and water bodies impacted by agriculture and aquaculture. Front. Microbiol..

[10] McNeilly O, Mann R, Hamidian M, Gunawan C (2021). Emerging concern for silver nanoparticle resistance in *Acinetobacter baumannii* and other bacteria. Front. Microbiol..

[11] He WY (2021). Clonal spread of *Escherichia coli* O101: H9-ST10 and O101: H9-ST167 strains carrying *fosA3* and *bla*_CTX-M-14_ among diarrheal calves in a Chinese farm, with Australian *Chroicocephalus* as the possible origin of *E. coli* O101: H9-ST10. Zool. Res..

[12] Contrepois M, Bertin Y, Pohl P, Picard B, Girardeau J-P (1998). A study of relationships among F17 a producing enterotoxigenic and non-enterotoxigenic *Escherichia coli* strains isolated from diarrheic calves. Vet. Microbiol..

[13] Begaud E, Mondet D, Germani Y (1993). Molecular characterization of enterotoxigenic *Escherichia coli* (ETEC) isolated in New Caledonia (value of potential protective antigens in oral vaccine candidates). Res. Microbiol..

[14] Wu R (2018). Fitness Advantage of *mcr*-1-bearing IncI2 and IncX4 plasmids in vitro. Front. Microbiol..

[15] Paveenkittiporn W, Kamjumphol W, Ungcharoen R, Kerdsin A (2020). Whole-genome sequencing of clinically isolated carbapenem-resistant *Enterobacterales* harboring *mcr* genes in Thailand, 2016–2019. Front Microbiol.

[16] Li R (2021). Comprehensive genomic investigation of coevolution of *mcr* genes in *Escherichia coli* strains via nanopore sequencing. Glob. Chall..

[17] Rozwandowicz M (2018). Plasmids carrying antimicrobial resistance genes in *Enterobacteriaceae*. J. Antimicrob. Chemother.

[18] Li W (2021). *mcr* expression conferring varied fitness costs on host bacteria and affecting bacteria virulence. Antibiotics (Basel).

[19] Meinersmann RJ (2019). The biology of IncI2 plasmids shown by whole-plasmid multi-locus sequence typing. Plasmid.

[20] Tansawai U (2021). Emergence of *mcr-*3-mediated IncP and IncFII plasmids in Thailand. J. Glob. Antimicrob. Resist..

[21] Vines J (2021). Transmission of similar *mcr*-1 carrying plasmids among different *Escherichia coli* lineages isolated from livestock and the farmer. Antibiotics (Basel).

[22] Snesrud E, McGann P, Chandler M (2018). The Birth and demise of the ISApl1-*mcr*-1-ISApl1 composite transposon: The vehicle for transferable colistin resistance. MBio.

[23] Snesrud E (2017). Analysis of serial isolates of *mcr-*1-positive *Escherichia coli* reveals a highly active ISApl1 transposon. Antimicrob. Agents Chemother..

[24] Wang Y (2017). Prevalence, risk factors, outcomes, and molecular epidemiology of *mcr-*1-positive *Enterobacteriaceae* in patients and healthy adults from China: An epidemiological and clinical study. Lancet Infect. Dis..

[25] Yang QE (2020). Compensatory mutations modulate the competitiveness and dynamics of plasmid-mediated colistin resistance in *Escherichia coli* clones. ISME J..

[26] Alba P (2018). Molecular epidemiology of *mcr*-encoded colistin resistance in *Enterobacteriaceae* from food-producing animals in Italy revealed through the EU harmonized antimicrobial resistance monitoring. Front. Microbiol..

[27] Partridge SR, Kwong SM, Firth N, Jensen SO (2018). Mobile genetic elements associated with antimicrobial resistance. Clin. Microbiol. Rev..

[28] Carroll AC, Wong A (2018). Plasmid persistence: Costs, benefits, and the plasmid paradox. Can. J. Microbiol..

[29] Sandegren L, Lindqvist A, Kahlmeter G, Andersson DI (2008). Nitrofurantoin resistance mechanism and fitness cost in *Escherichia coli*. J. Antimicrob. Chemother..

[30] San Millan A (2009). Multiresistance in *Pasteurella multocida* is mediated by coexistence of small plasmids. Antimicrob. Agents Chemother..

[31] Vogwill T, MacLean RC (2015). The genetic basis of the fitness costs of antimicrobial resistance: A meta-analysis approach. Evol. Appl..

[32] Singhal N, Kumar M, Kanaujia PK, Virdi JS (2015). MALDI-TOF mass spectrometry: An emerging technology for microbial identification and diagnosis. Front. Microbiol..

[33] Rebelo AR (2018). Multiplex PCR for detection of plasmid-mediated colistin resistance determinants, *mcr-*1, *mcr*-2, *mcr*-3, *mcr*-4 and *mcr-*5 for surveillance purposes. Euro Surveill.

[34] Khine NO (2020). Multidrug resistance and virulence factors of *Escherichia coli* harboring plasmid-mediated colistin resistance: *mcr*-1 and *mcr-*3 genes in contracted pig farms in Thailand. Front. Vet. Sci..

[35] Wang M (2003). Plasmid-mediated quinolone resistance in clinical isolates of *Escherichia coli* from Shanghai, China. Antimicrob. Agents chemother..

[36] Bolger AM, Lohse M, Usadel B (2014). Trimmomatic: A flexible trimmer for Illumina sequence data. Bioinformatics.

[37] Wick RR, Judd LM, Gorrie CL, Holt KE (2017). Unicycler: Resolving bacterial genome assemblies from short and long sequencing reads. PLoS Comput. Biol..

[38] McArthur AG (2013). The comprehensive antibiotic resistance database. Antimicrob. Agents Chemother..

[39] Gupta SK (2014). ARG-ANNOT, a new bioinformatic tool to discover antibiotic resistance genes in bacterial genomes. Antimicrob. Agents Chemother..

[40] Chen L, Zheng D, Liu B, Yang J, Jin Q (2016). VFDB 2016: Hierarchical and refined dataset for big data analysis–10 years on. Nucleic Acids Res..

[41] Seemann T (2014). Prokka: Rapid prokaryotic genome annotation. Bioinformatics.

[42] Hua X (2021). BacAnt: A Combination annotation server for bacterial DNA sequences to identify antibiotic resistance genes, integrons, and transposable elements. Front. Microbiol..

[43] Pal C, Bengtsson-Palme J, Rensing C, Kristiansson E, Larsson DG (2014). BacMet: Antibacterial biocide and metal resistance genes database. Nucleic Acids Res..

[44] Alikhan NF, Petty NK, Ben Zakour NL, Beatson SA (2011). BLAST Ring Image Generator (BRIG): Simple prokaryote genome comparisons. BMC Genomics.

[45] Sullivan MJ, Petty NK, Beatson SA (2011). Easyfig: A genome comparison visualizer. Bioinformatics.

[46] Katoh K, Standley DM (2013). MAFFT multiple sequence alignment software version 7: Improvements in performance and usability. Mol. Biol. Evol..

