# Author Correction: Comparative genomic analysis of Colistin resistant *Escherichia coli* isolated from pigs, a human and wastewater on colistin withdrawn pig farm

**DOI:** 10.1038/s41598-024-66994-y

**Published:** 2024-07-10

**Authors:** Nwai Oo Khine, Thidathip Wongsurawat, Piroon Jenjaroenpun, David J. Hampson, Nuvee Prapasarakul

**Affiliations:** 1 Center of Excellence in Diagnosis and Monitoring of Animal Pathogens (DMAP), Department of Veterinary Microbiology, Faculty of Veterinary Science, Chulalongkorn University, Bangkok, Thailand; 2 Division of Bioinformatics and Data Management for Research, Research Group and Research Network Division, Faculty of Medicine Siriraj Hospital, Mahidol University, Bangkok, Thailand; 3 School of Veterinary Medicine, Murdoch University, Perth, WA Australia

Correction to: *Scientific Reports* 10.1038/s41598-023-32406-w, published online 29 March 2023

The Funding section in the original version of this Article was incomplete.

Funding

“Funding for the study was received from the Second Century Fund (C2F batch 5/2022), Chulalongkorn University, Thailand, Royal Golden Jubilee Ph.D. (RGJPHD) program, The Agricultural Research Development Agency (ARDA) [CRP6205031110], the CHE-TRF Senior Research Fund (RTA6280013), Thailand Science Research and Innovation, and Pathogen Bank, Faculty of Veterinary Science, Chulalongkorn University, Bangkok, Thailand and the 90th Anniversary of Chulalongkorn University Scholarship”.

now reads,

Funding

“Funding for the study was received from the Second Century Fund (C2F batch 5/2022), Chulalongkorn University, Thailand, Royal Golden Jubilee PhD (RGJPHD) program, The Agricultural Research Development Agency (ARDA) [CRP6205031110], by the 2567-Fundamental Fund, Thailand Science Research and Innovation (TSRI), Chulalongkorn University (FOOD), Pathogen Bank, Faculty of Veterinary Science, Chulalongkorn University, Bangkok, Thailand, and the 90th Anniversary of Chulalongkorn University Scholarship”.

The original Article has been corrected.

