# Supplementary material for: Comparative genomic analysis of Colistin resistant Escherichia coli isolated from pigs, a human and wastewater on colistin withdrawn pig farm
Source: Sci Rep. 2023 Mar 29;13:5124. doi: 10.1038/s41598-023-32406-w (PMC10060365; doi:10.1038/s41598-023-32406-w)
Supplement: Supplementary file 1 — Supplementary Information. [file 41598_2023_32406_MOESM1_ESM.pdf]

Supplementary table 1. Heavy metal resistance genes detected in six MCRPE isolates

Strains	Year	Source	Location	Heavy metal resistance genes
CP52E	2017	Human	Chromoso-me	Copper (*pcoA*, *pcoB*, *pcoC*, *pcoD*, *pcoR*, *pcoS*, *pcoE*), Zinc (*zntA)*,<br>Silver (*silE*, *silS*, *silC*, *silF*, *silB*, *silA*, *silP*)
CPE35	2017	Pig	Chromoso-me	Zinc (*zntA)*
CPWW7	2017	Wastewat er	Chromoso-me<br>Plasmid	Zinc (*zntA)*<br>Mercury (*merR_Ps*, *merT*, *merC*),
CPF6	2018	Pig	Chromoso-me	Copper (*pcoE*, *pcoS*, *pcoD*, *pcoC*, *pcoB*, *pcoA*), Zinc (*zntA*),<br>Silver (*silP*, *silB*, *silF*, *silC*, *silS*, *silE*)
CPWW CT	2018	Wastewat er	Chromoso-me	Copper (*pcoA*, *pcoB*, *pcoC*, *pcoD*, *pcoS*, *pcoE*), Zinc (*zntA)*,<br>Silver (*silE*, *silS*, *silC*, *silF*, *silB*, *silA*, *silP*)
CPA 1200	2019	Pig	Chromoso-me	Copper (*pcoS*, *pcoD*, *pcoC*, *pcoB*, *pcoA*), Zinc (*zntA)*, Silver (*silP*, *silA*, *silB*, *silF*, *silC*, *silS*, *silE*)

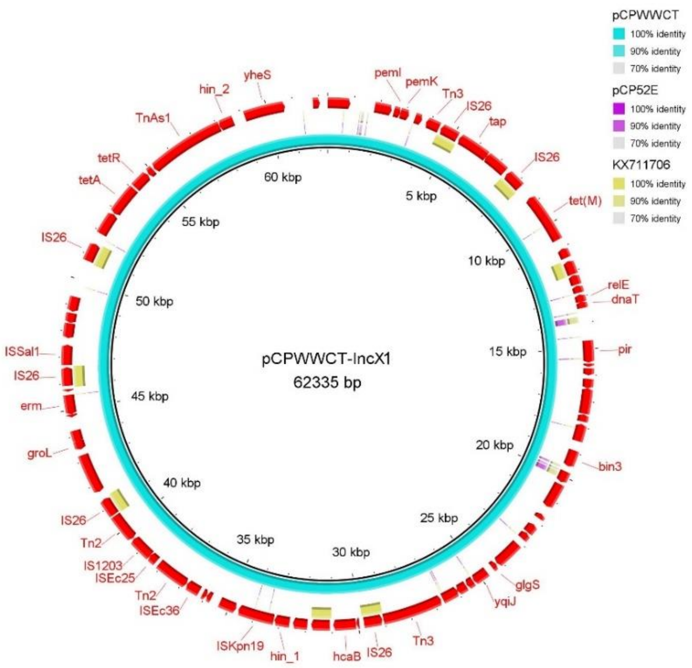

Supplementary figure 1. Sequence alignment of pCPWWCT-IncX1 (wastewater, 2018) plasmid with pCP52E (human, 2017) and the reference plasmid pCSZ4 (GenBank no. KX711706). The outer circle with red arrows denotes annotation of the plasmid pCPWWCT-IncX1.

Supplementary table 2. Information about reference sequences used in this study

No	Accession number	Strain	Species	Country origin	Plasmid name	Year	Source
1	KU994859.1	KP81	*E. coli*	Belgium	pKP81-BE,	2016	Pig
2	AP018353.1	E769	*E. coli*	Vietnam	pVE769	2018	Food
3	MF679146.1	Not provided	*E. coli*	Hong Kong	pBJ114-141	2013	Human
4	CP049119.1	EC931	*E. coli*	China	pEC931_1	2017	Human
5	EU935738.1	D	*E. coli*	UK	pEK516	2008	Human
6	LSAP01000114.1	CMCEC_NDM_2	*E. coli*	India	CMCEC_NDM_2 plasmid	2014	Human
7	MH329656.1	92944	*E. coli*	China	p92944-CTXM	2018	Hospital
8	MT449722.1	PN24	*E. coli*	Thailand	PN24 plasmid pPN24	2020	Duck
9	MT449721.1	PN156	*E. coli*	Thailand	pPN156	2020	Human
10	MT449720.1	PN42,	*E. coli*	Thailand	pPN42	2020	Human
11	CP042621.1	NCYU-26-73-6	*E. coli*	Taiwan	pNCYU-26-73	2017	Pig
12	CP077064.1	Q4552	*E. coli*	France	pECQ4552_	2020	Pig
13	CP029748.1	2016C-387	*E. coli*	USA	pMCR1-PA	2016	Human
14	CP042642.1	NCYU-24-74	*E. coli*	Taiwan	pNCYU-24-74-4	2017	Pig
15	NZ_CP069658.1	O89m:H10	*E. coli*	Poland	pMUB-MIN12-1	2018	Human
16	NZ_CP015913	210205630	*E. coli*	USA	pSLy1	2016	Pig
17	NZ_CP026644.1	FORC_082	*E. coli*	South Korea	pFORC82_3	2017	Chicken meat
18	NZ_CP018118	MRSN346638	*E. coli*	Germany	pMRSN346638	2016	Human
19	NZ_CP018106	MRSN352231	*E. coli*	Germany	pMR0716_mcr1	2016	Human
20	KY829117	WCHEC1604	*E. coli*	China	pMCR_WCHEC1604	2015	Sewage
21	KY075654	Lishui142	*E. coli*	China	pLishui142-1	2017	Human
22	MG557851	Not provided	*E. coli*	Thailand	PN21	2017	Chicken
23	MN232187	Not provided	*E. coli*	China	GD16-131	2019	Chicken
24	MF774183.1	SHP16	*E. coli*	China	pHNSHP16	2017	Pig

25	KX254342	JS-61	*E. coli*	Hong Kong	pECJS	2016	Pig
26	KU761326	SZ02	*E. coli*	China	SZ02 plasmid pmcr1	2016	Human blood
27	MN106912	TA9	*E. coli*	China	pTA9	2019	Duck meat
28	KY012275	27COE1	*E. coli*	China	pEc_27COE18	2016	Human
29	KX034083	A31-12	*E. coli*	China	pA31-12	2016	Not provided
30	MN746291	GN2982	*E. coli*	Ecuador	p25	2019	Dog
31	CP051226	SCZE5	*E. coli*	China	pSCZE4	2020	Pig
32	OK323956	ECP81	*E. coli*	Thailand	pJS021	2021	Pig
33	AP017622.1	MRY15-131	*E. coli*	Japan	pMRY15-131_2	2016	Cattle
34	KP347127.1	SHP45	*E. coli*	China	pHNSHP45	2016	Pig
35	KY693674.1	OM97	*E. coli*	UAE	pOM97-mcr	2017	Human
